# Acute Right Ventricular Dysfunction Secondary to Hereditary Angioedema Exacerbation

**DOI:** 10.7759/cureus.15336

**Published:** 2021-05-30

**Authors:** Abbas Shahmohammadi, Kathryn M Burtson

**Affiliations:** 1 Division of Pulmonary, Critical Care, and Sleep Medicine, University of Florida, Gainesville, USA; 2 Department of Internal Medicine, Wright-Patterson Air Force Base/Wright State University, Dayton, USA

**Keywords:** hereditary angioedema, right ventricular dysfunction, troponin

## Abstract

This is a case report of a 31-year-old woman with past medical history of hereditary angioedema (HAE) who developed acute right ventricular dysfunction. The patient presented to the emergency department with complaints of acute abdominal pain and swelling. Her electrocardiogram demonstrated sinus tachycardia and T wave inversion in leads V1-V3, otherwise without findings suggestive of ischemia. Troponin was elevated at 1.83 ng/mL. A transthoracic echocardiogram showed normal left ventricular function with ejection fraction of 65-70%, but the right ventricle (RV) was dilated and severely hypokinetic and there was moderate tricuspid regurgitation. Patient was managed symptomatically for her HAE exacerbation. Her abdominal swelling resolved, troponins continued to trend down, and she was discharged home after three days. A follow up echocardiogram done six months later demonstrated normal RV function.

## Introduction

The physiology and anatomy of the right ventricle (RV) is much different than its neighbor the left ventricle. Factors that influence the RV include preload, afterload, and left ventricular function [1,2]. Increased pressure and or volume leads to RV failure resulting in ischemia and RV dilation [1,2]. The unique crescent-shaped anatomy of the RV itself results in poor tolerance of acute elevations in afterload [1]. Hereditary angioedema (HAE) is a rare and life-threatening disease. The prominent symptoms of hereditary angioedema include acute airway swelling, abdominal swelling, and recurrent gastrointestinal colic. Abdominal attacks are an incredibly painful and common presentation in approximately 80% of HAE patients, often leading one to seek medical attention [3]. We present the case of a young female who presented with acute hereditary angioedema exacerbation with abdominal swelling and was found to have right ventricular dysfunction.

## Case presentation

**Figure 1:**
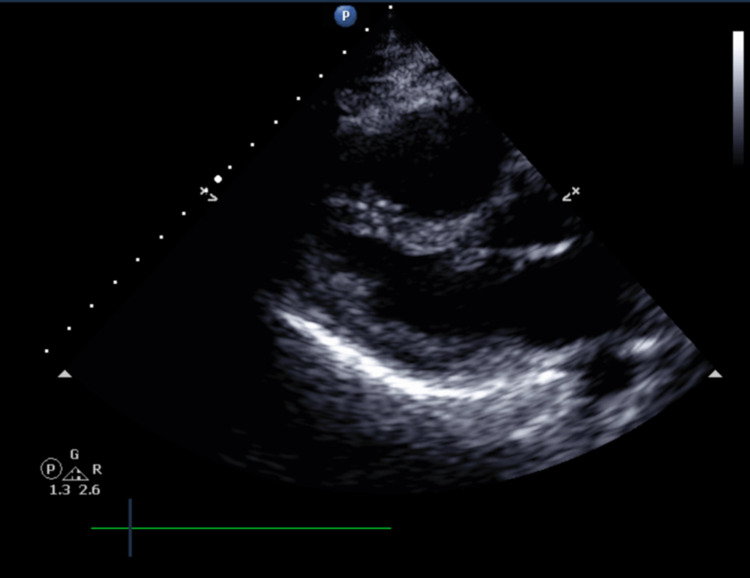
Transthoracic echocardiogram showed that right ventricle was dilated and severely hypokinetic with moderate tricuspid regurgitation

A 31-year-old female with history of hereditary angioedema (HAE) presented with acute abdominal pain with swelling and distension. The patient had a history of HAE and her exacerbations resulted in significant abdominal pain, swelling and, distension. On exam, she appeared in moderate distress. Vitals were significant for heart rate of 120. Abdominal exam revealed significant distension, hypoactive bowel sounds, tenderness to palpation, and guarding without any localization. Lungs were clear to auscultation. Electrocardiogram demonstrated sinus tachycardia with new T wave inversions in leads V1-V3, otherwise there were no acute findings suggestive of ischemia. Troponin obtained was elevated at 1.83 ng/mL. Chest X-ray showed no acute pathology. A transthoracic echocardiogram showed normal left ventricular function with ejection fraction of 65-70%, but the right ventricle was dilated and severely hypokinetic with moderate tricuspid regurgitation [Figure 1].

**Figure 2:**
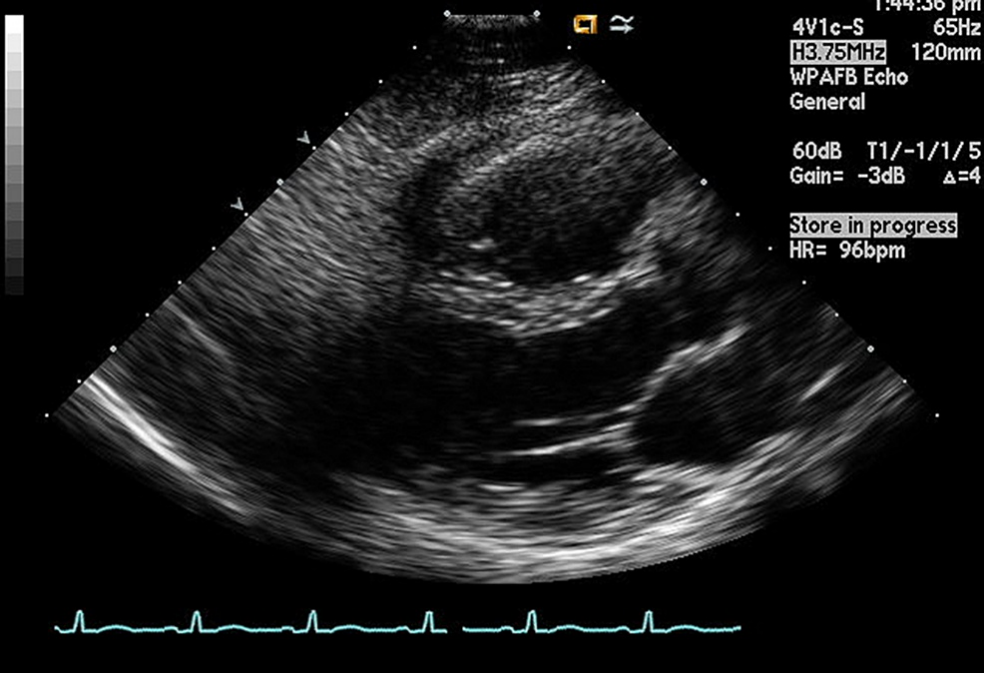
Right ventricle after recovery: follow-up echocardiogram done six months later showed normal right ventricle function

Baseline echocardiogram completed one month prior to admission was normal. She underwent ventilation perfusion scan which was low probability for PE. Cardiology was consulted, acute coronary syndrome with RV infarct was thought to be unlikely, and thus cardiac catheterization was not pursued. Patient was managed supportively with volume resuscitation and symptomatically for her HAE exacerbation. Her abdominal swelling resolved and troponins continued to trend down and she was discharged home after three days. A follow-up echocardiogram done six months later showed normal RV function [Figure 2]. 

## Discussion

This case demonstrates a rare etiology of RV dysfunction secondary to increased intra-abdominal pressure from acute flare up of HAE. Hereditary angioedema is an inborn autosomal dominant disease due to a deficiency of C1 inhibitor (C1INH) or a dysfunctional protein [4]. C1INH inhibits both catalytic functions of kallikrein and activated factor XII [4]. Removal of this inhibition results in compliment activation and elevation of bradykinin [5]. During HAE exacerbations, C1INH deficient patients demonstrate markedly elevated levels of bradykinin [4]. This massive bradykinin release promotes many symptoms of HAE, fostering increased capillary permeability, vasodilation, edema, and fluid extravasation in affected organs [3,5].

Manifestations of intestinal angioedema include nausea, vomiting, diarrhea, abdominal pain, and abdominal distention from ascites [3]. Increased intra-abdominal pressure can cause increased intra-thoracic pressure resulting in direct cardiac compression [6]. Moreover, increased pulmonary vascular resistance due to compression of lung parenchyma can result in increased RV afterload [6].

Factors that influence right ventricular function include preload, afterload, and left ventricular function [1,2]. The anatomy and physiology of the RV itself results in poor tolerance to acute increase in afterload [1]. The crescentic shaped anatomy of the RV results in poor tolerance of elevations in afterload [1]. The free wall of the RV is predominantly perfused by the right coronary artery, receiving equivalent flow during diastole and systole [7]. Even in the absence of atherosclerotic coronary disease, imbalances in myocardial supply and demand may develop [1]. Increased pressure and or volume leads to RV failure resulting in ischemia and RV dilation [1,2]. Tricuspid regurgitation occurs secondary to annular dilatation and poor leaflet co-optation. Rising filling pressures and diastolic dysfunction are compounded by tricuspid regurgitation [7]. Treatment of RV dysfunction entails removal of the underlying cause as well as the maintenance of systemic arterial pressure and biventricular contractility [8]. Preload augmentation with volume resuscitation can partially ameliorate intra-abdominal pressure-induced increases in afterload [6].

## Conclusions

In this case, the patient’s HAE exacerbation led to acute abdominal swelling. This is an unusual case report of acute HAE causing abdominal distension leading to RV dysfunction. Maintaining a high index of suspicion of RV failure as a consequence of an abdominal HAE exacerbation may improve outcomes in such cases.

## References

[1] Matthews JC, McLaughlin V (2008). Acute right ventricular failure in the setting of acute pulmonary embolism or chronic pulmonary hypertension: a detailed review of the pathophysiology, diagnosis, and management. Curr Cardiol Rev.

[2] Mebazaa A, Karpati P, Renaud E, Algotsson L (2004). Acute right ventricular failure--from pathophysiology to new treatments. Intensive Care Med.

[3] Rubinstein E, Stolz LE, Sheffer AL, Stevens C, Bousvaros A (2014). Abdominal attacks and treatment in hereditary angioedema with C1-inhibitor deficiency. BMC Gastroenterol.

[4] Longo D, Fauci A, Kasper D, Hauser S, Jameson J, Loscalzo J (2012). Harrisons Manual of Medicine, 18th Edition. https://www.mhebooklibrary.com/doi/book/10.1036/9780071808309.

[5] Cheatham ML (2009). Abdominal compartment syndrome: pathophysiology and definitions. Scand J Trauma Resusc Emerg Med.

[6] Nzeako UC (2010). Diagnosis and management of angioedema with abdominal involvement: a gastroenterology perspective. World J Gastroenterol.

[7] Voelkel NF, Quaife RA, Leinwand LA (2006). Right ventricular function and failure: report of a National Heart, Lung, and Blood Institute working group on cellular and molecular mechanisms of right heart failure. Circulation.

[8] Stephanazzi J, Guidon-Attali C, Escarment J (1997). [Right ventricular function: physiological and pathophysiological features]. Ann Fr Anesth Reanim.

